# Editorial

**Published:** 1849-03

**Authors:** 


### THE MEDICAL EXAMINER.

### PHILADELPHIA, MARCH, 1849.

### THE SUMMER MEDICAL SCHOOLS OF PHILADELHHIA.

(From a Correspondent.)

At this season the professors in our medical colleges are doubtless rejoicing at the approaching close of their yearly labours, and the thousand students who have filled our halls of science are equally elated, we are sure, at the prospect of the speedy emancipation that awaits them. But although the lessons of these eminent instructors shall for the present cease, and the throng of their pupils disperse,—yet in the interval between the conclusion of one and the commencement of another winter session, medical science is not without its interpreters in this city, nor, we are happy to add, without numerous and diligent disciples.

For thirty years there has been one or more institutions in which all the ordinary branches of a medical education have been taught, from April or May until November in every year, and the number of students who have annually availed themselves of their advantages has seldom, we believe, fallen below one hundred. The quality of the instruction imparted may be inferred from the fact that of the fourteen professors in the two largest colleges of Philadelphia, all but three were at some time connected with these summer schools.

The traditions of summer medical teaching may safely be said to live in undiminished vigour, for at no previous date have more intelligence, industry, and experience, been devoted to this important pursuit. In several respects, indeed, and particularly in the means of illustrating lectures by apparatus, specimens, models, drawings, &c., and by clinical observation, the summer schools of the present day have greatly the advantage of their predecessors. They are, besides, advancing constantly in the road of improvement; for they are stimulated by a spirit of generous rivalry which urges them to compete for intelligent students by superior advantages, instead of degrading both themselves and medical science by flattering the indolent, and those who have yet to learn that cheap knowledge is of all cheap things the most worthless. Such a spirit indicates something more than personal good feeling, and fairness of dealing among the parties; it offers the strongest possible security that the good of the pupils is the primary and paramount object of the lecturers. A glance at the names of these gentlemen, nearly all of whom are favorably known to the profession, will strengthen this assurance. They are as follows:

Philadelphia Association for Medical Instruction.—*Anatomy*, J. M. Allen, M. D.; *Physiology*, F. G. Smith, Jr., M. D.; *Materia Medica and Therapeutics*, Francis West, M. D.; *Medical Chemistry*, Robert Bridges, M. D.; *Diseases of Children*, J. Forsyth Meigs, M. D.; *Institutes and Practice of Surgery*, J. M. Wallace, M. D.; *Pathology and Practice of Medicine*, Alfred Stillé, M. D.; *Obstetrics and Diseases of Women*, David H. Tucker, M. D.

Medical Institute of Philadelphia.—*Anatomy*, John Neill, M. D.;

*Materia Medica and Therapeutics,* JOHN J. REESE, M. D.; *Obstetrics and Diseases of Women,* WM. BYRD PAGE, M. D.; *Surgery,* HENRY H. SMITH, M. D.; *General and Special Internal Pathology,* MEREDITH CLYMER, M. D.; *Physiology,* JOSEPH LEIDY, M. D.—"Dr. HORNER will assist in the lectures on Anatomy."

PENNSYLVANIA HOSPITAL.—*Clinical Medicine,* W. PEPPER, M. D., and W. W. GERHARD, M. D.; *Clinical Surgery,* EDWARD PEACE, M. D., and GEORGE FOX, M. D.

WILLS' HOSPITAL.—*Clinical Ophthalmic Surgery* by ISAAC PARRISH, M. D., and GEORGE FOX, M. D.

It is evident from this enumeration that all, and more than all, of the branches taught in the colleges are fully treated of by the summer lecturers, and yet the student, instead of spending five or six hours daily in the lecture room, is subjected to only one-half that amount of confinement. He can, moreover, pursue such courses only as he may select; and, if he prefer to attend but a part of the lectures in one school, or to take the ticket of one or more lecturers in each school, he is still at liberty to do so. Both the "Institute" and the "Association" have been careful to arrange the lecture hours so as to enable their classes to follow the *daily* clinical instruction at the Pennsylvania Hospital, the lessons given at Wills' Hospital upon diseases of the eye, and the Dispensary consultations and operations at the University, and the Jefferson College.

The opportunity of studying bed-side medicine forms one of the chief attractions and merits of the summer schools of Philadelphia. All experienced teachers are agreed that the principal defect in the education of medical students in this country is the superficial acquaintance they obtain of the phenomena and treatment of disease. They are well grounded in Anatomy, Chemistry and Materia Medica; they acquire a tolerable knowledge of Physiology, and learn all that they can be expected to learn of the practice of Medicine, Surgery and Obstetrics, from the most eminent professors of these departments. But very few amongst them are competent, upon graduation, to determine the nature of a given case of disease, to direct its treatment, or to comprehend, or even detect, the changes of structure revealed by dissection. They have not, in general, even such a familiarity with the physiognomy of disease as will put their investigations upon the right track when they commence the practice of their profession.

Nothing, so much as this defect, is calculated to paralyze the efforts of the young physician, and dishearten him not only in his first, but in all his subsequent combats with disease. For there is much, very much, in hospital practice which may be readily learned from a competent instructor, but which none but the most gifted minds can acquire when left to their unaided resources. To such as intend to reside in a large city or populous town, the mischief is less serious, because on every side the counsel and assistance of experienced and skilful men are to be procured; but to the vast majority of practitioners, to those who must fix their residence in thinly peopled districts, and, living almost entirely isolated as regards medical association, must depend altogether upon themselves, the mischief is incalculable, and what is worse, without remedy. The time and opportunity for retrieving their loss are gone forever.

The attempts which some of our colleges have made to supply the want of clinical instruction by means of public consultations and operations in the amphitheatre, are in themselves praiseworthy; but none more than they who have at the time felt satisfied with such lessons, have subsequently owned their inadequacy to take the place of hospital instruction,—the substance, of which these demonstrations are merely the shadow. A summer's course in Philadelphia affords the best possible opportunity for attending to this essential branch of medical education; for while the student is daily trained in the didactic and demonstrative branches of his science, and has, therefore, its principles constantly fresh in his memory, he, with equal frequency, is permitted to learn at the bed-side that without which all the rest of his knowledge would be almost useless. He there learns to recognize diseases, and to distinguish them from one another; he learns how to ascertain their history, and the value of their symptoms; to watch their changes from day to day through every stage from invasion to convalescence, and how to meet the special indications which they present, with appropriate combinations, and an opportune application of remedies. His clinical studies, in fact, stand him in the stead of many years of experience, by the variety of cases to which they relate, and still better by affording him a safe guide and example where he must have groped and doubted if left to himself.

A notion has very generally prevailed that our summer schools are severally attached to some of the medical colleges of this city, playing the part, as it were, of nurseries to these latter, and consequently that the Lecturers of the former are little else than *répetiteurs*, or recapitulators of the Professors. How so erroneous an idea could have become accredited, it is difficult to conjecture. But certain we are that the

Professors themselves could never have given countenance to so injurious a supposition. To them it must of course be gratifying to know that their students, during the summer, are listening to the same truths which they themselves teach, but presented in another form and manner than their own; and they cannot but acknowledge that the fitness of the Lecturers for their office is much better shown by an original mode of teaching, than by a slavish imitation of themselves either in doctrine or style. We are certain that the insinuation referred to could never have proceeded from the students in the summer-schools; for these gentlemen would scarcely be attracted by the diluted reproduction of a course which they must of necessity hear twice in the winter season,—they naturally prefer what is original, even if reputed to be inferior, for in such they cannot fail at least to find something novel either in statement or opinion.

It may be objected that this double and somewhat divergent instruction on the same subject may confuse the student's mind, or disturb his faith in the dicta of his Professor. The objection is more specious than sound. The lectures of both classes of teachers are composed of facts and opinions; the former must be much the same in each; the latter, if antagonistic, merely illustrate to the student what it is well that he should learn betimes—*opinionem delet dies.* His solid acquirements will be none the less, because he finds that different explanations may be given of the same phenomena.

But although the relation of the summer-schools to the colleges is not one of dependence, nor yet that of copyists, it is one of subordination in the hierarchy of public instruction, a position which is viewed with pride and satisfaction by the Lecturers, as one second only in dignity and usefulness to the Professorship itself. For it does not admit of doubt that of those students who attend the summer-schools, a larger proportion graduate with distinction, and practice with success, than of any equal number who attend the winter courses alone. It is true that a great part, if not all of the merit thus obtained is apt to be referred, by strangers, to the school in which the successful student graduates; but *he* is never backward in attributing a liberal share of the credit to his summer-teachers, and they, in their turn are amply rewarded by seeing the excellent fruits of their comparatively unappreciated labour. That they rest contented with playing the part of auxiliaries, and do not repine at seeing their light merged in the brightness of the Colleges' reputation, proceeds from a conviction of the real eminence of their position; for it depends upon themselves alone whether they shall continue to hold it, or assume one of rivalry towards the winter-schools, by procuring the right to confer medical degrees. It is scarcely doubtful that a capable and honest school or schools, possessing such powers, and adopting the summer instead of, or along with the winter term, would attract a large number of students; and it is very certain that neither capacity nor honesty are necessary to ensure the grant of such powers in these days of liberal legislation. For ourselves, we should earnestly deprecate any such step, for we believe that the true interests of medicine in this country are best to be promoted by restricting far within its present limits the right of conferring degrees, while the avenues to medical knowledge are at the same time multiplied to whatever extent the wants of the community may require. "Free trade" in teaching can work no harm, if it is not coupled with a corresponding license to stamp goods of every quality with one and the same mark; but an indiscriminate grant to impose the title of Doctor of Medicine, amounts to a permission to commit forgery with impunity.

We repeat, then, that the vocation of our summer lecturers is equally honourable and important. Nothing but ignorance of its great utility, as well in maturing and storing the student's mind, as in giving him direct preparation for practice, can account for the fact that so small a proportion of the whole number of medical students avail themselves of its advantages. For their information, and for that of the gentlemen who are intrusted with the direction of their professional education, these pages were written. We are persuaded that they will find the subject one of greater moment than they have supposed, and, it may be added, one that is yearly growing in consequence; for with the determination of the medical public to insist upon a more complete education of students than heretofore, and the difficulties of extending and enlarging the winter courses with all the desirable completeness and rapidity, the summer schools acquire an additional importance. Indeed, if properly supported, they would go very near to curing the defects in our system of education, of which complaint is so widely and so justly made.

---

SYDENHAM SOCIETY'S WORKS.

The first volume of this most useful Society's publications for the year 1848–9 has probably, by this time, reached our shores. From unavoidable circumstances, it has been detained longer than usual. It is Rhazes on the Small Pox, edited by Dr. Greenhill, of Oxford—a learned and industrious physician—in every way competent to the task. "The next volumes," says the Secretary, Dr. James Risdon Bennett, in a letter to Dr. Dunglison, which we have, seen, "will be *Hippocrates*, Vol. I., and *Sydenham's Life and Works*, in English, Vol. I. We shall also give you a fourth for the year, viz.: *Rokitansky's Pathological Anatomy*, Vol. I. Both in amount and variety, therefore, I trust, that we shall abundantly satisfy our trans-Atlantic friends." For the sum of *five dollars*, paid to the Honorary Local Secretary, and the expenses of transmission and duty, amounting in all to about *six dollars and a half*, four handsome and valuable volumes are placed in the hands of subscribers here. The volumes for the 5th year of the Society's existence were—one of *Paulus Ægineta ; Feuchtersleben's Medical Psychology*, *Microscopical Researches of Schwann and Schleiden*, and a volume of *Memoirs of the French Academy of Surgery*.

In a country where there are so many professional readers, the immense labour—of the most disinterested kind too—voluntarily undertaken by many of the most distinguished physicians and surgeons of Great Britain, composing the board of management of the Sydenham Society, can scarcely fail to to be appreciated, and exhibited by the increasing number of names in their annual catalogue from this side of the Atlantic.

---

DELEGATES.

We give below a list of those who have been selected from the College of Physicians of Philadelphia, and from the Medical Societies of the counties of Philadelphia and Alleghany, as delegates to our State Medical Society, which meets at Reading, in April, and to the American Medical Association, to be held at Boston in May next. Other societies may have made similar selections, but as yet the names of the delegates have not reached us. We hope no society will neglect the election of delegates to our National Association, because we consider it essential to the success of the movements of that body, that its meetings should be participated in by as large a number of the profession as possible. Let the representation be full; let harmony and good feeling control the conduct of each delegate, and the cause of medical reform must prevail. In union there is strength, and the members of the association should meet, disposed to compromise conflicting opinions; for however they may differ as individuals in regard to the best means of effecting medical reform, there is not one of them who does not desire sincerely the improvement of the profession of which he is a member. This, we are convinced, is the wish of *all*. Why then cannot all come forward in a spirit of good fellowship and aid in the reformation of those abuses which are said to exist in our system of education. In the performance of their duties, we trust, the members of the National Medical Association will remember that *every change, is not reform*. It is better to improve than to destroy our present system; but these improvements, introduced with caution and judgment, should always be modified to suit the peculiar condition of things by which we are surrounded in this country.

---

*Alleghany County (Pa.) Medical Society.*—At a meeting of the Medical Society of Alleghany County, Pa., the following officers were elected for the present year:—*President*, Dr. Joseph P. Gazzam; *V. Presidents*, Drs. J. Brooks, R. B. Mowry; *Cor. Sec.*, Dr. Samuel Dilworth; *Rec. Secs.*, Drs. A. M. Pollock, John S. Irwin; *Treasurer*, Dr. George D. Bruce; *Censors*, Drs. Joseph P. Gazzam, J. Brooks, E. G. Edrington; *Board of Examiners*, Dr. Bobert Snyder, T. McKennon, William McK. Morgan.

Drs. Robert Snyder and Wm. McK. Morgan were elected delegates to the State Medical Convention, to meet at Reading.

Dr. Joseph P. Gazzam was elected delegate to the National Medical Convention, to meet at Boston.

Drs. Wm. Addison, J. Brooks and D. McNeal were appointed a committee to confer with a committee from the City Councils upon the subject of the cholera.

---

At a meeting of the Philadelphia College of Physicians held February 6th, 1849, the following were elected Delegates to the American Medical Association:—George B. Wood, Francis West, Alfred Stillé, George W. Norris, Isaac Hays, D. Francis Condie, Henry Bond, Samuel Jackson (late of Northumberland), George Fox, Gouverneur Emerson.

---

At a meeting of the Philadelphia County Medical Society held January 30th, 1849, the following were elected Delegates to the State Society:—H. S. Patterson, T. Hobson, F. G. Smith, Isaac Hays, W. Jewell, T. F. Betton, T. S. Reed.

And the the following were elected Delegates to the American Medical association :—J. H. Yardley, W. H. Klapp, W. Maybery, M. M. Reeve, W. B. Page, H. Gibbons, T. F. Betton.

---

At a meeting of the Philadelphia County Medical Society held January 30th, 1849, the following resolutions were adopted :

Resolved, That the Society has heard with great satisfaction, that a renewed effort is making in the Legislature of Pennsylvania, to procure the enactment of a law for the registration of Births, Marriages, and Deaths.

Resolved, That believing such a law adapted to promote, in a high degree, the health, and therefore the prosperity and happiness of the people, as well as to guard more effectually the rights of the citizens, this Society would unite with the National and local Societies which have already addressed the Legislature upon the subject, in urging upon that honorable body the early enactment of a Registration Law.

---

A notice of the transactions of the College of Physicians and of several Introductory Lectures will appear in our next number.

---

It will be seen by reference to our advertising sheet, that Mr. J. C Turnpenny offers for sale a complete collection of articles intended as illustrations to a course of Lectures on Materia Medica. The medicines and the dried plants have been selected with the greatest care, and the paintings, executed by Wm. McIlvaine, are beautiful and faithful representations of most of the medicinal plants. We would advise those engaged in teaching, to examine this collection, if they feel disposed to supply themselves with handsome illustrations of the Materia Medica.

---

In the notice of *Morton's Human Anatomy*, in the last number of the Examiner, the names of the Publishers were omitted ; they are *Messrs. Grigg & Elliot*, No. 14 N. Fourth Street.

